# Supplementary material for: Bayesian Covariate-Dependent Circadian Modeling of Rest-Activity Rhythms
Source: Data Sci Sci. Author manuscript; Available in PMC 2026 Jul 3. (PMC13327678; doi:10.1080/26941899.2025.2474943)
Supplement: supplementary [file NIHMS2182813-supplement-supplementary.pdf]

Supplementary Material to “Bayesian Covariate-Dependent Circadian Modeling of Rest-Activity Rhythms”

Beniamino Hadj-Amar[a], Vaishnav Krishnan[b] and Marina Vannucci[a]

[a]Department of Statistics, Rice University, Houston, TX; [b]Neurology, Neuroscience, and Psychiatry & Behavioral Sciences, Baylor College of Medicine, Houston, TX.

ARTICLE HISTORY
Compiled February 26, 2025

This supplementary material provides further details about the simulation study and actigraphy data analysis, including MCMC convergence diagnostics and trace plots, posterior predictive checks for representative individuals, and subject-specific parameter estimates, highlighting the variability and individual-specific nature of activity patterns.

A. Simulation Study

This section provides further details about the Simulation Study presented in Section 3 of the manuscript.

A.1. Convergence Diagnostic

MCMC chains were run for 2,000 iterations, excluding the first 1,000 as the burn-in period, leaving 1,000 iterations for inference. The convergence of the MCMC chains was evaluated using the Gelman-Rubin convergence diagnostic, with R-hat statistics not exceeding 1.01, indicating no pathological behavior. A representative trace plot of the parameter vector $\boldsymbol{\eta}_a$, consisting of the linear coefficients governing amplitude projected in the l_1-ball space, is displayed in Figure S1. These traces show good mixing and accurately target the true generating values. Parameters different from zero consistently mix away from zero, while parameters equal to zero are almost always projected to assume exact zero values.

A.2. Posterior Predictive

In Figure S2, we present an additional graphical posterior predictive check, showcasing observations alongside 100 draws from the estimated posterior predictive distribution for four representative individuals. These results demonstrate that the model effectively captures the dynamics of the data.

CONTACT: B.H. Email: bh44@rice.edu

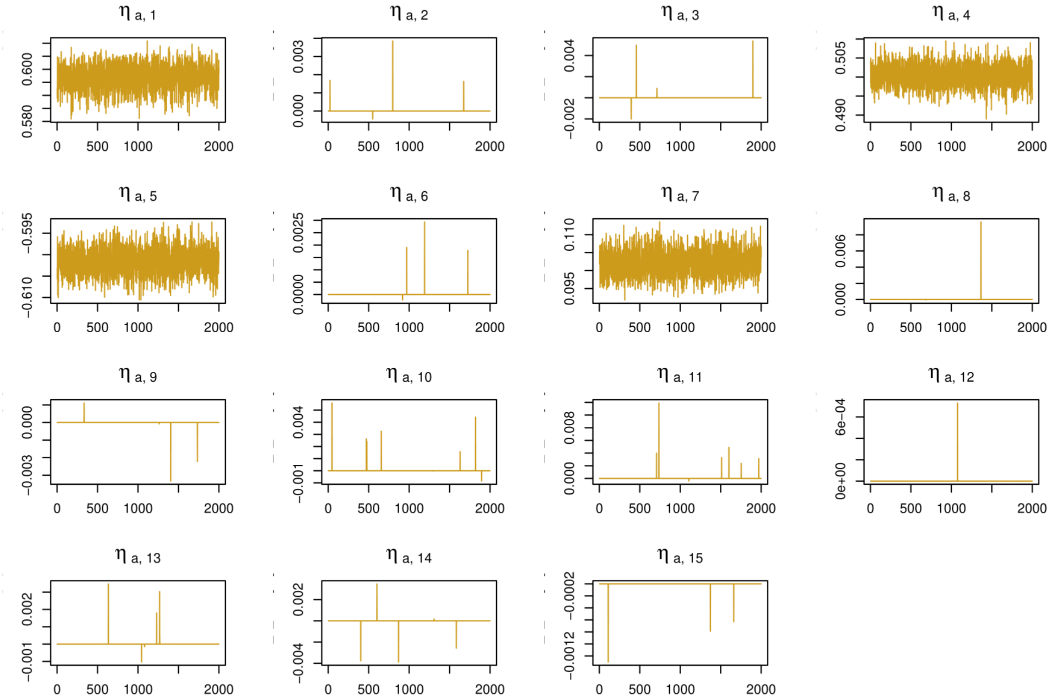

Figure S1.: Simulation Study. A representative trace plot of the parameter vector $\boldsymbol{\eta}_a$, consisting of the linear coefficients governing amplitude projected in the l_1-ball space.

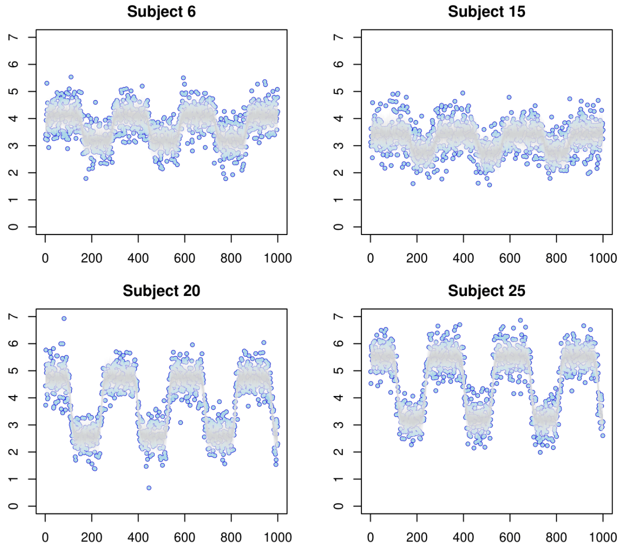

Figure S2.: Simulation Study. Graphical posterior predictive check, showcasing observations alongside 100 draws from the estimated posterior predictive distribution for four representative individuals.

A.3. Out-of-sample predictive performance

We investigate the out-of-sample predictive performance of our proposed approach by comparing it with CALC Blasso, the standard cosinor model applied independently to each subject, and the extended cosinor approach (RAR) also run on a per-subject basis. Specifically, for each sample size ($T_i = 500$ and $T_i = 1000$), we train the models on the observed data and simulate an additional 25% of observations to form a test set. In line with our simulation study (Section 3), we consider several noise settings with residual standard deviation $\sigma \in \{0.5, 1.0, 1.5, 2.0\}$. Out-of-sample prediction accuracy is quantified using the log-transformed RMSE and RMAE, computed between the true generating signal and the predicted signal on the test set. Table S1 summarizes these results, showing that our CALC l_1 model consistently achieves lower (i.e., better) log-RMSE and log-RMAE values compared to CALC Blasso, RAR, and the standard cosinor model, thereby demonstrating its superior predictive performance.

		$T_i = 500$						$T_i = 1000$			
		$\sigma = 0.5$	$\sigma = 1.0$	$\sigma = 1.5$	$\sigma = 2.0$			$\sigma = 0.5$	$\sigma = 1.0$	$\sigma = 1.5$	$\sigma = 2.0$
RMSE	CALC l_1	-0.566	-0.549	-0.549	-0.549	RMSE	CALC l_1	-0.514	-0.497	-0.518	-0.52
	CALC BLasso	1.79	1.81	1.83	1.87		CALC BLasso	1.78	1.79	1.80	1.81
	RAR	1.94	2.00	2.14	2.30		RAR	2.17	2.17	2.24	2.28
	cosinor	2.44	2.45	2.48	2.52		cosinor	2.42	2.43	2.44	2.45
RMAE	CALC l_1	-0.549	-0.540	-0.542	1.88	RMAE	CALC l_1	-0.536	-0.527	-0.539	-0.54
	CALC BLasso	1.80	1.81	-0.536	-0.527		CALC BLasso	1.76	1.77	1.78	1.80
	RAR	1.86	2.06	2.24	2.39		RAR	2.08	2.17	2.27	2.34
	cosinor	2.44	2.46	2.49	2.53		cosinor	2.40	2.41	2.43	2.45

Table S1.: Simulation Study. Out-of-sample predictive performance metrics for CALC l_1, CALC Blasso, RAR, and the standard cosinor model. Log-transformed RMSE and RMAE are reported for two sample sizes ($T_i = 500$ and $T_i = 1000$) across four noise levels ($\sigma \in \{0.5, 1.0, 1.5, 2.0\}$). Lower values indicate better predictive accuracy.

B. Actigraphy Data: Subject-specific Parameter Analysis

We provide further results from the application of our model to the actigraphy data. Figure S3 displays subject-specific estimates for the parameters $a(\boldsymbol{X}, \boldsymbol{\eta}_a)$ (amplitude), $\phi(\boldsymbol{X}, \boldsymbol{\eta}_\phi)$ (phase), $\boldsymbol{\beta}$ (scale), $\boldsymbol{\alpha}$ (rest-activity ratio), $\boldsymbol{m}$ (baseline activity level), and $\boldsymbol{\sigma}$ (residual standard deviation). These plots offer a comprehensive visual representation of how each parameter varies across subjects, highlighting the diversity and individual-specific nature of the activity patterns.

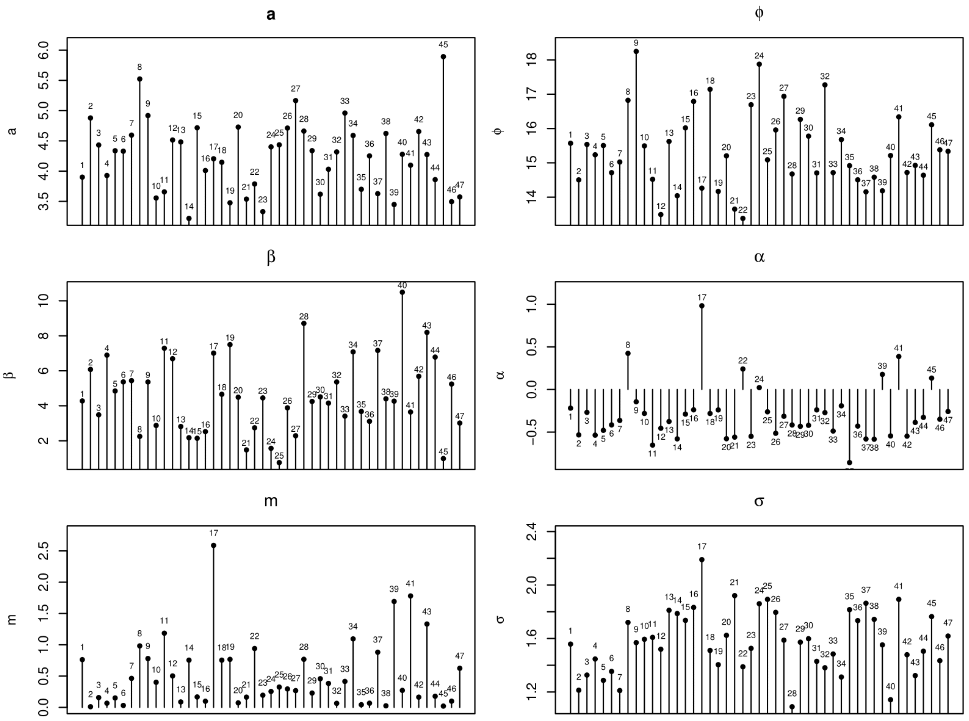

Figure S3.: Subject specific estimate of parameters $a(\boldsymbol{X}, \boldsymbol{\eta}_a)$, $\phi(\boldsymbol{X}, \boldsymbol{\eta}_\phi)$, $\boldsymbol{\beta}$, $\boldsymbol{\alpha}$, $\boldsymbol{m}$, and $\boldsymbol{\sigma}$.
